# Energy efficient cluster-based routing protocol for WSN using multi-strategy fusion snake optimizer and minimum spanning tree

**DOI:** 10.1038/s41598-024-66703-9

**Published:** 2024-07-22

**Authors:** Le Yang, Damin Zhang, Lun Li, Qing He

**Affiliations:** College of Big Data & Information Engineering, Guizhou University, Guiyang, 550025 China

**Keywords:** Computer science, Information technology

## Abstract

In recent years, the widespread adoption of wireless sensor networks (WSN) has resulted in the growing integration of the internet of things (IoT). However, WSN encounters limitations related to energy and sensor node lifespan, making the development of an efficient routing protocol a critical concern. Cluster technology offers a promising solution to this challenge. This study introduces a novel cluster routing protocol for WSN. The system selects cluster heads and relay nodes utilizing the multi-strategy fusion snake optimizer (MSSO) and employs the minimum spanning tree algorithm for inter-cluster routing planning, thereby extending the system’s lifecycle and conserving network energy. In pursuit of an optimal clustering scheme, the paper also introduces tactics involving dynamic parameter updating, adaptive alpha mutation, and bi-directional search optimization within MSSO. These techniques significantly increase the algorithm convergence speed and expand the available search space. Furthermore, a novel efficient clustering routing model for WSN is presented. The model generates different objective functions for selecting cluster heads and relay nodes, considering factors such as location, energy, base station distance, intra-cluster compactness, inter-cluster separation, and other relevant criteria. When selecting cluster heads, the fuzzy c-means (FCM) algorithm is integrated into MSSO to improve the optimization performance of the algorithm. When planning inter-cluster routing, the next hop node is selected for the relay node based on distance, residual energy, and direction.The experimental results demonstrate that the proposed protocol reduces energy consumption by at least 26.64% compared to other cluster routing protocols including LEACH, ESO, EEWC, GWO, and EECHS-ISSADE. Additionally, it increases the network lifetime of WSN by at least 25.84%, extends the stable period by at least 52.43%, and boosts the network throughput by at least 40.99%.

## Introduction

WSN are autonomous networks consisting of several small, portable, or stationary sensor nodes. To achieve real-time monitoring of the surrounding region, sensors collect data from the environment and transmit it through network routing to the central gathering center or base station(BS)^1^. WSN are particularly appealing because of their low cost, high fault tolerance, low power consumption, and multifunction capabilities. These days, WSN are increasingly being used in advanced communications, the Internet of Things (IoT), intelligent medical services, agricultural management, and other industries, thanks to the progress of cloud-based computing^2^.

In WSN, each node serves as both a sensor and a router, with limited computational capability, storage capacity, and communication capacity. Furthermore, many WSN applications utilize sensor nodes placed in hostile locations, which raises the challenge and cost of replacing malfunctioning nodes^3^. Therefore, in order to prolong network lifetime and enable wireless nodes to operate for extended periods without requiring battery replacement, energy efficiency must be considered when implementing a network routing protocol^4^. It is vital to remember that the majority of operational WSNs assess physical phenomena such as temperature, pressure, humidity, or the location of objects,so the sensing data from adjacent areas may be closely related in this instance. Furthermore, data transmission requires more energy than data processing^5^. Consequently, in order to achieve the goal of energy conservation, it is necessary to aggregate adjacent sensor data before transmission to reduce communication costs in the network.

**Figure 1:**
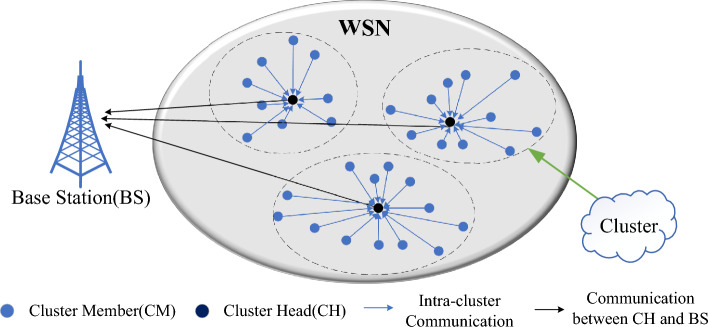
Cluster architecture in wireless sensor networks.

In this specific case, the use of cluster technology increases network scalability and reduces power consumption of routing protocols^6^. As shown in Fig. 1, the sensor nodes are arranged into clusters in the cluster architecture, and the sensor nodes with lower energy are used for sensing tasks. The sensor data is then transmitted to the cluster head (CH) over a shorter distance. The CH sends the aggregated data to the BS after filtering out redundant data from the cluster members (CM). The clustering approach helps to reduce the substantial energy expenditure associated with direct data transfer between each node and the BS, thereby preventing network failure. By reducing the total energy consumption and distributing the energy usage among nodes over the network’s lifespan, it extends the network’s longevity and enhances energy efficiency. Furthermore, it may reduce packet conflicts and channel congestion to enhance network throughput under heavy load, which is crucial for the development of high-performance networks and advanced industrial applications^7^.

The process of selecting an appropriate CH within a network and establishing a stable cluster to minimize energy consumption has been demonstrated to be an NP-hard problem^8^, traditional clustering technology may not be capable of achieving the optimal solution. As research into evolutionary computing has progressed, many scholars have begun to use different heuristic algorithms such as particle swarm optimization (PSO), genetic algorithm (GA), ant colony algorithm (ACO), differential evolution algorithm (DE) and artificial bee colony algorithm (ABC) in an attempt to select a reasonable CH, and then get a better cluster solution^9,10^. However, when the existing cluster routing protocols relying on heuristic algorithms try to obtain the optimal cluster scheme in WSN, the algorithms tend to converge prematurely and reach local optimum, which will lead to the increase of network energy consumption and the shortening of network lifetime. In addition, most of the existing protocols do not consider the best route between the cluster head and the base station in the data transmission stage, which consumes a lot of energy when the cluster head transmits data directly to the base station, resulting in network performance damage.

Considering the above issues, this paper creatively proposes a new cluster routing protocol. The proposed protocol first combines the snake optimizer based on multi-strategy fusion with the constructed new objective function to establish a stable and reliable cluster scheme, and uses the minimum spanning tree algorithm to design inter-cluster routing. Finally, the overall energy consumption of the network is reduced, the network life cycle is prolonged, and the stability and communication quality of the network are improved.

**Figure 2:**
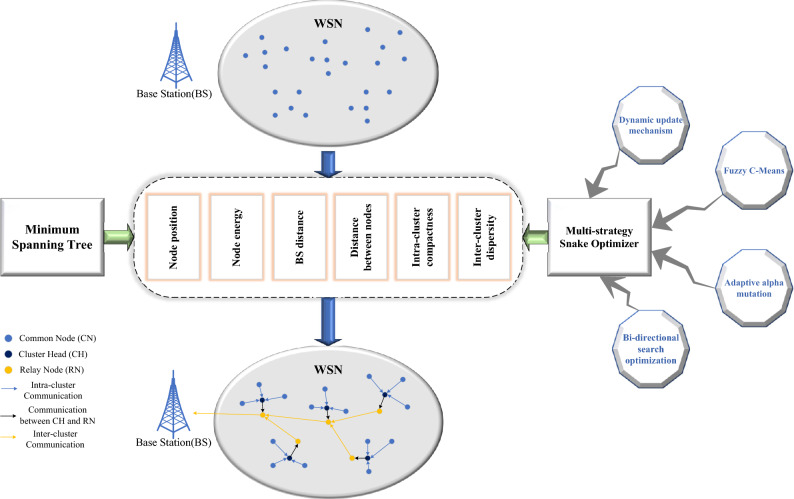
Central focus of the study.

Figure 2 illustrates the central focus of this study. The primary research components of this investigation are outlined as follows:

- This paper creatively proposes an efficient cluster routing protocol for WSN, which significantly enhances the energy level of the system. It greatly reduces the energy consumption generated by each protocol operation, effectively prolongs the service life of WSN, and makes it suitable to meet the application requirements of industrial production, agriculture, Internet of things and other fields.
- A novel energy-saving cluster routing model is designed, which can accurately reflect the real situation of WSN and significantly improve the network performance. In this model, the CH node is responsible for collecting aggregated cluster data, and the relay node (RN) is responsible for sharing data transmission tasks with the CH to balance the load, and transmits data to the BS through reasonable inter-cluster routing. In addition, this study considers key factors such as node location, node energy, base station distance, intra-cluster compactness, inter-cluster dispersion, and node directionality to construct different objective functions for selecting CH and RN and designing inter-cluster routing.
- This study selects CH and RN using a multi-strategy fusion snake optimizer (MSSO). In comparison to other heuristic algorithms, MSSO has a stronger ability to break out of the local optimum and can accomplish more sufficient search in the search space in order to obtain a better cluster scheme. This can be due to the introduction of dynamic parameter update, bidirectional search optimization and adaptive alpha mutation strategy. This work also incorporates the FCM algorithm into the MSSO structure for cluster head node selection, hence enhancing the algorithm’s convergence speed and optimization performance. Furthermore, this study constructs a link weight model and applies the minimal spanning tree technique to produce the best inter-cluster routing from the relay node to the base station when the relay node transmits data.
- A new simulation experiment is carried out to evaluate the effectiveness and scalability of the protocol. Compared to other existing cluster routing protocols including LEACH, ESO, EEWC, GWO and EECHS-ISSADE, the proposed protocol has obvious advantages in residual energy, energy balance, network lifetime, network stability period, and network communication quality. Specifically, the energy consumption of the proposed protocol is reduced by at least 26.64 %, the network lifetime of WSN is increased by at least 25.84 %, the stable period is increased by at least 52.43 %, and the network throughput is increased by at least 40.99 %.

This paper is organized as follows: Section "Introduction" provides an introduction to the necessary background. Section "Related work" presents the related work. The newly designed WSN model is introduced in Sect. "Model definition". Section "Proposed protocol" introduces the proposed protocol. In Sect. "Experimental results and analysis", the proposed protocol is compared with several existing cluster routing protocols through simulation experiments. Section "Conclusion" concludes the paper by summarizing the work conducted and offering insights into future research directions.

## Related work

### Classical techniques

Cluster-based routing protocols have been widely designed to improve the energy efficiency and lifetime of WSN^11^, among which LEACH protocol is one of the most famous clustering protocols^12^. It pioneers the concept of cluster, works in a round manner, and rotates CH between sensor nodes based on a predetermined probability to achieve the purpose of balancing network energy consumption. However, the randomness of CH selection makes some inappropriate nodes in LEACH protocol may be selected as CH, such as nodes with low residual energy or nodes located at the edge of the network. At the same time, there may be problems of uneven distribution of CH and unstable number of CH^13^. In this case, a poor cluster will be formed, which accelerates the death of network nodes. In addition, the CH in the LEACH protocol directly transmit data to the BS. When the BS is far away from the network, node overload may occur, causing the CH to die too quickly. Aiming at the defects of LEACH protocol, many researchers have made corresponding improvements on the basis of the protocol^14,15^.

LEACH-C is proposed to enhance network performance, the centralized clustering algorithm is used by the protocol to build clusters, and in order to improve cluster quality, CH is distributed throughout the entire network. When choosing CH, the energy component is taken into account simultaneously. The nodes with higher residual energy have a greater probability of being selected as CH. The LEACH-C protocol, however, still selects CH with a high degree of randomness, and the uneven distribution of CH leads to an excessive consumption of network energy. To establish a more balanced cluster and increase the network lifetime, the BCDCP protocol is proposed^16^, inter-cluster routing is implemented to prevent CH from sending data directly to the BS, and each cluster has approximately the same number of members to minimize CH overload. The HEED protocol is proposed to reduce the transmission cost between nodes^17^, elements like node degree and node residual energy are taken into consideration while selecting the CH. The CH sends data to the BS through multi-hop communication. However, the CH nodes in the protocol still consume a lot of energy, causing some nodes to die prematurely. The LPLL-LEACH protocol^18^ quantitatively analyzes and calculates the optimal number of cluster heads. The election probability of cluster heads and the manner in which normal nodes join cluster heads are computed by taking into account several criteria. Furthermore, the protocol uses the forwarding function to determine the best next hop cluster head for forwarding data. Simultaneously, it facilitates direct communication between isolated nodes and the base station, thereby extending the node death time and enhancing data transmission efficiency.

### Metaheuristic techniques

The optimal solution to balance the network’s energy usage is tough to find in conventional cluster routing protocols, and it obviously deviates from the credible answer. In this case, many classical metaheuristic algorithms are used in WSN to obtain reliable cluster solutions because of their search ability, robustness and adaptability, so as to reduce the energy consumption during network operation^19,20^. Rao et al.^21^ proposed an energy-saving cluster protocol PSO-ECHS based on particle swarm optimization algorithm. The protocol uses the position mapping strategy to form an efficient coding scheme, and considers the factors such as the distance within the cluster, the distance between the node and the BS, and the residual energy of the node to design the objective function. It clearly performs better than the classical protocol, but it ignores the load of CH and doesn’t take the best transmission path from the CH to the BS into account. Pal et al.^22^ proposed an energy-efficient clustering routing protocol EEWC based on binary genetic algorithm. The objective function of the protocol includes three elements: cluster compactness, cluster separation and CH number. The EEWC protocol balances the energy consumption of the network and improves network longevity. Attea et al.^23^ considered the ratio of intra-cluster distance to inter-cluster distance and the number of CH to design the objective function, and used the binary differential evolution algorithm to obtain a reliable cluster scheme, which maintained a good balance between network lifetime and reliability. With the goal to reduce the energy loss of nodes, Gülşah et al.^24^ took into account the distance between nodes as well as their residual energy, and used simulated annealing algorithm to minimize the energy loss of nodes. Zou et al.^25^ used the improved ant colony algorithm to adaptively construct inter-cluster routing according to the dynamic characteristics of the network, which improved the efficiency of data transmission, but the protocol did not fully consider how to form a reasonable cluster. Mann et al.^26^ improved the development ability and convergence of the artificial bee colony algorithm through the Student ’s-t distribution, and then obtained the best CH set in the network based on the improved algorithm, which has good performance in data packet transmission, energy consumption, and lifecycle.

Benefiting from the rapid development of evolutionary computing, several new metaheuristic algorithms have been proposed to solve various optimization problems^27,28^. These algorithms have better performance than conventional algorithms. Researchers have begun to try to use new metaheuristic algorithms to obtain better clustering schemes in wireless sensor networks. Jaiswal et al.^29^ used the grey wolf optimizer (GWO) to select CH within the cluster according to different factors such as energy level, node degree, and BS distance. At the same time, forwarding nodes were selected based on the cost function to achieve multi-hop routing from CH to BS. The protocol’s over-reliance on the cluster’s original creation is an issue, though. Maheshwari et al.^30^ used the butterfly optimization algorithm (BOA) to select the best CH set according to various factors, and used the ant colony algorithm to obtain the route between the CH and the BS. It performs well in terms of network throughput and node survival time. Panimalar et al.^31^ first used the differential evolution strategy to improve the sparrow search algorithm, and then used the improved sparrow search algorithm (SSA) to solve the problem of CH selection in WSN. Afterward, they used the high search efficiency of the algorithm to obtain a better clustering scheme, saving network energy consumption. Solayan et al.^32^ proposed an energy-efficient routing protocol based on metaheuristic strategy, which integrates energy consumption, distance, throughput, packet transmission rate, path loss and other factors into a fitness function, and uses adaptive remora optimization algorithm (ROA) for CH selection. In addition to saving energy, the protocol extends the life of the network. Rani et al.^33^ proposed a routing technology based on fault-tolerant clusters, which selects CH for each cluster through a large escape optimization algorithm, and selects additional backup CH for each cluster to continue data transmission when CH fails. The protocol also introduces a special node called Aggregator for data aggregation to reduce the energy consumption caused by data aggregation in each CH. In addition, the protocol also uses the improved particle swarm optimization algorithm to estimate the inter-cluster routing of CH to reduce the communication overhead. Kaur et al.^34^ proposed an energy-efficient clustering protocol based on artificial fish swarm algorithm (EAFSCP) inspired by the collective behavior of fish swarm. It has a good performance in enhancing network performance and prolonging network lifetime. However, the energy efficiency of the EAFSCP protocol is limited due to the fact that there are few factors to consider when selecting CH and the transmission route from CH to BS is not designed.

### Combined techniques

In terms of solution quality, metaheuristic-based clustering protocols outperform conventional techniques. Nevertheless, it frequently takes longer for these methods to converge on the optimal answer. Recently, researchers have coupled metaheuristic algorithms with additional techniques to achieve better routing performance.

Nigam et al.^35^ used the particle swarm optimization algorithm combined with K-means clustering to create an optimized cluster. After the initial cluster was established, different fitness functions were used to select the appropriate nodes in the cluster according to the distance from the BS. The protocol prolong the network’s life cycle by rotating between being the cluster head, although it is heavily dependent on the initial cluster’s formation. Pradeep et al.^36^ proposed a cluster-based routing protocol for wireless body area networks. The grey wolf optimizer is improved by Q-learning to improve the convergence of the algorithm, and the improved grey wolf optimizer is used to select the best CH according to distance, energy, transmission power and other factors to maximize the energy utilization of the cluster. Similarly, fewer factors are considered in the selection of CH and the heavy burden of CH is also a problem in this protocol.

Since fuzzy logic systems respond quickly in real time, they are often used in conjunction with metaheuristic algorithms for particular types of wireless networks. Fanain et al.^37^ used fuzzy logic combined with whale optimization algorithm to select CH and select forwarding CH for CH according to three factors : node location, node residual energy, and node degree, to avoid the situation that far-distance CH directly send data to the base station consumes a lot of energy. However, the overload of forwarding CH may affect the performance of the protocol. Memarian et al.^38^ proposed the TSFIS-GWO protocol for adaptive real-time routing in WBAN. This protocol combines the Takagi-Sugeno fuzzy inference system with a simpler structure and easy parameter optimization with the grey wolf optimizer. The Takagi-Sugeno fuzzy rules of the TSFIS model are optimized using grey wolf optimizer in the offline phase based on variables including network longevity, dependability, and path loss. Ultimately, the network stability is improved by building dependable network routing based on various node and link statuses using the modified fuzzy system. Aryai et al.^39^ proposed a swarm intelligence multi-objective fuzzy protocol (SIMOF) as an adjustable routing protocol in WBAN. The two stages of SIMOF are the automated rule adjustment (ARA) using the whale optimization algorithm and the fuzzy inference system (FIS). During the offline phase, the whale optimization algorithm obtains the best Mamdani fuzzy rules in the FIS. The FIS then selects the suitable cluster head using a variety of fuzzy criteria based on residual energy, distance, reliability, path loss, and predicted energy consumption. The protocol performs well in real time and is quite flexible. The goal of Esmaeili et al.’s^40^ hybrid metaheuristic-driven machine learning routing protocol (MDML-RP) is to maximize routing selection in WBANs to accomplish load balancing, energy efficiency, and network lifetime extension.The principal objective of MDML-RP is to gather a large dataset of optimized routes that whale optimization algorithm has generated for a variety of network configurations and applications. Subsequently, it uses SVR to identify the behavioral patterns of whale optimization algorithm during the route construction process. Ultimately, it applies the modified MDML-RP model to real-time routing. Aryai et al.^41^ presented a new routing model (CHM), capable of choosing a sensible CH in WBAN. The appropriate CH is chosen in CHM using a variety of fuzzily defined criteria, including residual energy, path loss, destination distance, and history of becoming a CH. Furthermore, the protocol automatically modifies the model’s hyperparameters using a genetic method. It has been demonstrated that the suggested CHM model lowers energy consumption and increases network longevity. The FFA-RF protocol^42^ is based on the fuzzy firefly algorithm (FFA) and random forest (RF). Through the simulation of FFA’s clustering behavior in various WSNs, this protocol gathers a comprehensive data set, which is then used to train and evaluate the RF model. In the end, the fuzzy priority factor of each network node is utilized to determine whether the trained model becomes a CH. The cluster protocol can handle online routing requests for new and unseen WSN instances and is very adaptive to particular applications.

The above-mentioned protocol, which fuses the speed of fuzzy systems with the high accuracy of metaheuristic algorithms, achieves good routing performance in specific networks. However, before a new network is deployed, an enormous amount of processing capacity is needed due to the high model complexity in the offline stage. and duration for optimizing routing methods. Furthermore, the aforementioned protocol overlooks the cluster head’s multi-hop scenario. In particular situations, isolated nodes may transmit data directly to the sink, which could cause certain nodes to die sooner than necessary. In addition, the original metaheuristic algorithm may exhibit premature convergence and fall into a local optimum when applied to the optimization of a particular model, which may cause the optimized model to deviate from the optimal situation.

### Our contributions against existing techniques

In summary, certain existing cluster protocols frequently take into account a limited amount of parameters when selecting CH, disregarding other factors that have an influence on the performance of cluster routing. While most existing cluster protocols do not consider inter-cluster routing planning for CH nodes, and some protocols designed for inter-cluster routing have the problem of excessive load on forwarding CH nodes.Furthermore, some protocols employ metaheuristic algorithms to optimize models and produce preferred cluster solutions, but they ignore the inherent flaws of the metaheuristic algorithm itself, which compromises the overall efficiency of network routing.

In view of the above challenges, this paper proposes an effective solution to overcome these problems. Firstly, this paper selects a relay node(RN) for each CH node to reduce the burden of the CH. When selecting the CH and the RN, the key factors such as node position, node energy, BS distance, intra-cluster compactness, and inter-cluster dispersion are taken into account to design a more effective objective function. In order to better optimize the objective function and obtain a more reasonable clustering scheme, this paper uses multi-strategy fusion snake optimizer to select CH and RN to avoid the impact on the final routing performance due to the algorithm falling into local optimum in the optimization process.The FCM is also embedded in multi-strategy fusion snake optimizer, which accelerate the convergence of the algorithm while ensuring the high quality of the cluster solution. In the data transmission process stage, each CH first sends its own aggregated data to its corresponding RN. Afterwards, based on various factors including distance, remaining energy and node direction, the minimum spanning tree method is applied to quickly determine whether the RN’s next hop node is another RN or base station, which reduces the probability that the RN is overloaded due to more forwarding tasks. Finally, the proposed scheme significantly improves the network cluster, and has good performance in node energy consumption, network lifetime, and communication quality.

**Table 1:** Description of key parameters in the equation.

Parameter	Description
*N*	Number of sensor nodes
*d*	Transmission distance
$l$	Packet length
$\varepsilon _{fs}$	Power amplification parameters of free space transmission
$\varepsilon _{mp}$	Power amplification parameters of multipath fading transmission
$\varepsilon _{da}$	Data aggregation parameter
$d_0$	Distance threshold
*p*	Probability of cluster heads
*K*	Number of cluster heads
$\omega$	The weight in the fitness function when selecting cluster head
$\lambda$	The weight in the fitness function when selecting relay node
$N_{all}$	Total population size of snakes
$N_m$	Number of male snakes
$N_f$	Number of female snakes
$S_{max}$	Population’s maximum bounds
$S_{min}$	Population’s minimum bounds
*T*	Total number of iterations
$th_{Q}$	Temperature threshold
$th_{T}$	Food threshold
$c_1$, $c_2$, and $c_3$	Fixed-value parameters in the snake optimizer
$\theta$, $\gamma$, and $\delta$	Characteristic parameters in alpha distribution
$\alpha$	Variable characteristic index in adaptive alpha mutation
$r_1$, $r_2$	Random numbers between 0 and 1 in bi-directional search optimization

## Model definition

### Network model

Assume that a wireless sensor network consisting of *N* sensor nodes is deployed in the monitoring area and that the monitoring data is routinely reported to the BS. The sensor nodes in the network are divided into CH, RN and common nodes (CN) according to their functions^43^, the network topology is shown in Fig. 2, and the following assumptions are made here:

1. Every sensor nodes are equal in terms of processing and storage capacity, and they are all likely to become a CH or RN.
2. The information observed by ordinary nodes is correlated, so the CH collects data from the cluster and aggregates them into fixed-length packets.
3. Every CH has a matching RN. In order to balance the CH’s load, the RN receives the aggregated data from the CH and transmits it to the BS in accordance with the inter-cluster routing.
4. BS and sensor nodes are fixed after deployment, and each node is assigned a unique index according to its location.
5. The initial energy of each sensor node is equal, and the network is a homogeneous network. The node declares death, terminates detecting monitoring, and stops taking part in communication when its energy runs out.
6. Every node measures surrounding parameters at a constant rate and periodically transmits data to the destination node.
7. Every node can adjust its transmission power based on the distance to the receiver, with a fixed number of power levels available.
8. Every node possesses a data link capable of managing all data traffic. Simultaneously, the connections between nodes exhibit symmetry. A node can determine the distance to another node solely by assessing the received signal power.
9. The BS has no energy limitations because it is powered outside.

### Energy model

**Figure 3:**
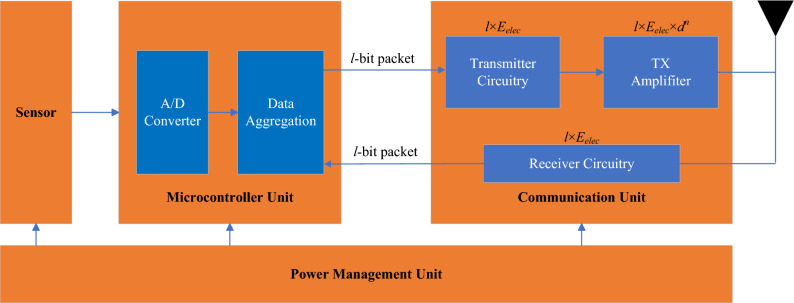
Sensor node components and radio energy model.

The sensor node’s components, including the microcontroller unit, communication unit, and power management unit, are depicted in Fig. 3. Here describes how energy consumption is taken into consideration in this paper: Both the energy consumed for data transmission and reception is included in the communication link’s energy consumption. Since the nodes in the network have data fusion capabilities, the amount of data that the CH needs to transmit is greatly reduced, thereby reducing the energy consumption of transmission within the network. However, it is still necessary to consider the energy consumption of data aggregation. The descriptions of key parameters are listed in Table 1. The total energy consumption in the network is as follows:

1 $$E_{TOTAL} = E_{TX} + E_{RX} + E_{DA}$$

In Eq. (1), $E_{TX}$ is the energy consumption of transmitting data, $E_{RX}$ is the energy consumption of receiving data, and $E_{DA}$ is the energy consumption of data fusion. Assuming that the transmission distance is $d$ and the packet length is $l$, the calculation methods of transmitting energy consumption, receiving energy consumption, and aggregation energy consumption are as follows:

2 $$\begin{aligned} E_{TX}(l, d)&= {\left\{ \begin{array}{ll} l\delta _{elec} + l\varepsilon _{fs}d^2 &{} \text {if } d < d_0 \\ l\delta _{elec} + l\varepsilon _{mp}d^4 &{} \text {if } d \ge d_0 \end{array}\right. } \end{aligned}$$

3 $$\begin{aligned} E_{RX}(l)&= l \times E_{elec} \end{aligned}$$

4 $$\begin{aligned} E_{DA}(l)&= l \times \varepsilon _{da} \end{aligned}$$

In Eq. (2), $\delta _{elec}$ represents the energy dissipated by the transmitter or receiver to process each bit of data, $\varepsilon _{fs}$ and $\varepsilon _{mp}$ are the power amplification parameters of free space transmission and multipath fading transmission, respectively. $\varepsilon _{da}$ is the data aggregation parameter, and $d_0$ is the distance threshold. If the transmission distance is less than $d_0$, the data is transmitted according to the free space model; otherwise, the multipath model is used to transmit data. The calculation method of $d_0$ is:

5 $$d_0 = \sqrt{\frac{\varepsilon _{fs}}{\varepsilon _{mp}}}$$

### Objective function

Assuming that the probability of CH is $p$, the WSN should be divided into $K$ different clusters, where $K = N_{alive} \times p$, $N_{alive}$ is the number of surviving nodes in the network. As mentioned above, each cluster includes a CH and its corresponding RN, and there are several CN. When selecting the CH, $\{CH_{1}, CH_{2}, ..., CH_{i}, ..., CH_{K}\}$ is defined as the set of CH, and $\{NCH_{1}, NCH_{2}, ..., NCH_{i}, ..., NCH_{N-K}\}$ is the set of non-cluster head nodes (NCH). When selecting RN, $\{RN_{1}, RN_{2}, ..., RN_{i}, ..., RN_{K}\}$ is defined as the set of RN, which is one-to-one corresponding to the CH set, and $\{CN_1, CN_2, ..., CN_i, ..., CN_{N-2K}\}$ is the set of CN. To achieve the goal of enhancing network performance, an effective objective function must be proposed and optimized to heuristically select the proper CH and RN.

#### Cluster head’s selection

Firstly, the distance between the node and the BS should be taken into account while selecting the CH because the distance determines the energy consumption when the node transmits to the BS. Thus, the nodes closer to the BS should be selected for the CH.

6 $$\begin{aligned} D_{CHtoBS} = \frac{1}{K}\sum _{i=1}^{K}\sqrt{\left( CH_{ix}-BS_{x}\right) ^2+\left( CH_{iy}-BS_{y}\right) ^2} \end{aligned}$$

where $D_{CHtoBS}$ is the average value of the sum of the distances from the cluster head to the base station, $K$ is the number of clusters in the network, $CH_{ix}$ and $CH_{iy}$ are the horizontal and vertical coordinates of the $i$ th CH, $BS_{x}$ and $BS_{y}$ are the horizontal and vertical coordinates of the base station.

Corresponding to $D_{CHtoBS}$, the distance from the NCH to the BS should also be considered in the objective function:

7 $$\begin{aligned} D_{NCHtoBS} = \frac{1}{N-K}\sum _{i=1}^{N-K}\sqrt{\left( NCH_{ix}-BS_{x}\right) ^2+\left( NCH_{iy}-BS_{y}\right) ^2} \end{aligned}$$

where, the $i$ th NCH’s horizontal and vertical coordinates are, respectively, $NCH_{ix}$ and $NCH_{iy}$. To make the CH’s position more reasonable, the objective function should also take into account the intra-cluster distance $D_{Intra}$ and the inter-cluster distance $D_{Inter}$:

8 $$\begin{aligned} D_{Intra}&= \frac{1}{K}\sum _{i=1}^K\sum _{j=1}^M\sqrt{\left( NCH_{jx}-CH_{ix}\right) ^2+\left( NCH_{jy}-CH_{iy}\right) ^2} \end{aligned}$$

9 $$\begin{aligned} D_{Inter}&= \frac{1}{K}\sum _{i=1}^K\sum _{j=1}^K\sqrt{\left( CH_{ix}-CH_{jx}\right) ^2+\left( CH_{iy}-CH_{jy}\right) ^2} \end{aligned}$$

In Eq. (8), the number of NCH in the cluster where the $i$ th CH located is denoted by *M*. $D_{Intra}$ represents the average intra-cluster distance of all clusters, reflecting the compactness within the cluster, while $D_{Inter}$ defined in Eq. (9) represents the average value of the sum of the distances between all CH, reflecting the separation of cluster distribution.

The position equalization coefficient is introduced in this paper to characterize the network’s candidate CH’s distribution state. The calculation method is as follows:

10 $$\begin{aligned} f_1 = \frac{D_{Intra} + D_{CHtoBS}}{D_{Inter} + D_{NCHtoBS}} \end{aligned}$$

In Eq. (10), if the closer the selected CH is to the BS, the smaller the distance between the NCH and the CH in each cluster, and the more uniform the distribution of each CH in the network, then the position of the CH is more balanced and the value of $f_1$ is smaller.

One further element that ought to be taken into account while selecting CH is the energy factor. This study describes the energy state of candidate CH using the energy equalization coefficient. The calculation method is as follows:

11 $$\begin{aligned} f_2 = \frac{\frac{1}{N-K}\sum _{j=1}^{N-K}E_{NCH}^r(j)}{\frac{1}{K}\sum _{i=1}^KE_{CH}^r(i)} \end{aligned}$$

where, CH residual energy is represented by $E_{CH}^r(i)$ and NCH residual energy by $E_{NCH}^r(j)$, respectively. If the average residual energy of the selected CH is higher, and it is heavier in the whole network, the value of $f_2$ is smaller.

In summary, this study employs a weighted method to evaluate the fitness of the candidate CH based on the position equalization coefficient and the energy equalization coefficient. The calculation method is as follows:

12 $$\begin{aligned} F_{CH} = \omega f_{1} + (1-\omega )f_{2} \end{aligned}$$

where $\omega$ is a constant ranging from 0 to 1, represents the weight. The CH set performs better when the fitness function $F_{CH}$ is smaller if the location distribution is more balanced and the residual energy of the selected CH set is higher.

#### Relay node’s selection

The RN is used to share data transmission tasks with the CH, that is, the RN receives the aggregated data packets of the CH and then sends the data packets to the BS to reduce the excessive energy consumption of the CH. This work employs a metaheuristic technique to allocate an appropriate RN to each selected CH, in comparison with the instability induced by random RN selection. The selection principle of RN is as follows:

Initially, the distance between the RN and the CH should be taken into account in order to minimize the energy consumption of the data shared by CH:

13 $$\begin{aligned} D_{RNtoCH}=\frac{1}{K}\sum _{i=1}^{K}\sqrt{\left( RN_{ix}-CH_{ix}\right) ^2+\left( RN_{iy}-CH_{iy}\right) ^2} \end{aligned}$$

where the $i$ th RN and its corresponding CH are represented by $RN_{i}$ and $CH_{i}$, respectively, whereas $D_{RNtoCH}$ indicates the average value of the sum of the distances between the RN and the corresponding CN.

To reduce the energy usage of the RN for data transmission, the objective function should further account for the distance between the RN and the BS and the distance between the RNs.

14 $$\begin{aligned} D_{RNtoBS}&=\frac{1}{K}\sum _{i=1}^{K}\sqrt{\left( RN_{ix}-BS_{x}\right) ^2+\left( RN_{iy}-BS_{y}\right) ^2} \end{aligned}$$

15 $$\begin{aligned} D_{RNtoRN}&=\frac{2}{K(K-1)}\sum _{i=1}^{K-1}\sum _{j=i+1}^{K}\sqrt{\left( RN_{ix}-RN_{jx}\right) ^2+\left( RN_{iy}-RN_{iy}\right) ^2} \end{aligned}$$

In Eq. (14), $D_{RNtoBS}$ represents the average distance from the RN to the BS, and in Eq. (15), $D_{RNtoRN}$ represents the average total distance between the RN. The smaller the $D_{RNtoBS}$ and $D_{RNtoRN}$, the closer the RN is to the base station, and the smaller the distance between the RN.

In order to avoid the RN’s position from being too near to the CH to interfere with the subsequent data transmission, it is also necessary to consider the distance between each CN and its associated CH as one of the contributing factors.

16 $$\begin{aligned} D_{CNtoCH}=\frac{1}{N-2K}\sum _{i=1}^{K}\sum _{j=1}^{M}\sqrt{\left( CN_{jx}-CH_{ix}\right) ^2+\left( CN_{jy}-CH_{{iy}}\right) ^2} \end{aligned}$$

where $D_{CNtoCH}$ is the average distance from all CN to their corresponding cluster heads.

To characterize the relay node’s position distribution, the position equalization coefficient is also suggested. The calculation method is as follows:

17 $$\begin{aligned} g_1=\frac{D_{RNtoCH}+D_{RNtoBS}+D_{RNtoRN}}{D_{CNtoCH}} \end{aligned}$$

In Eq. (17), the value of $g_1$ is smaller and the RN’s location is more balanced if the selected closer to the BS, each RN’s distance from its corresponding CH is smaller, and each RN is closer together.

The energy factor must also be considered when selecting the RN. The calculation method of the coefficient describing the energy status of the RN is as follows:

18 $$\begin{aligned} g_2=\frac{\dfrac{1}{N-2K}\sum \limits _{j=1}^{N-2K}E_{CN}^r(j)}{\dfrac{1}{K}\sum \limits _{i=1}^KE_{RN}^r(i)} \end{aligned}$$

In Eq. (18), $E_{RN}^r$ and $E_{CN}^r$ represent the residual energy of the RN and the CN respectively. If the average residual energy of the selected RN is higher, and the proportion of the whole network is relatively heavy except for the CH, the value of $g_2$is smaller. In summary, based on the position equalization coefficient and the energy equalization coefficient, this paper uses a weighted method to calculate the fitness of the candidate RN. The calculation method is as follows:

19 $$\begin{aligned} F_{RN}=\lambda g_1+(1-\lambda )g_2 \end{aligned}$$

where $\lambda$ is a constant ranging from 0 to 1, represents the weight. The RN set performs better when the fitness function $F_{RN}$ is smaller if the location distribution is more reasonable and the residual energy of the selected RN set is higher.

## Proposed protocol

**Figure 4:**
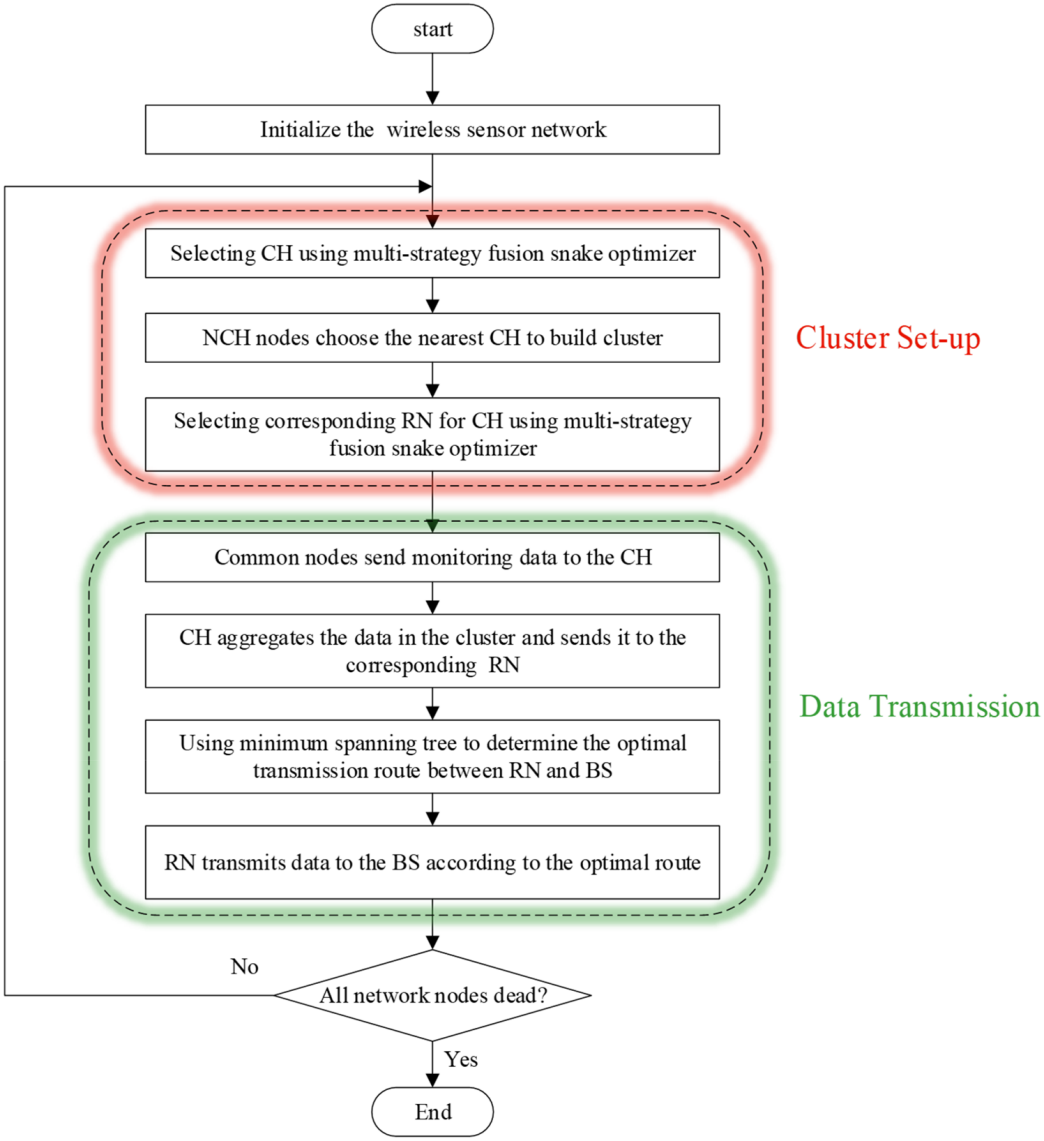
Flowchart of the proposed protocol.

The protocol proposed in this paper runs in rounds and repeats periodically in the network. The operation of each round includes two stages: cluster set-up stage and data transmission stage. A multi-strategy fusion snake optimizer (MSSO) is used to select the CH to establish a cluster during the cluster set-up stage. MSSO is also employed in each cluster to select the RN. The CH gathers data from CN in cluster during the data transmission phase, then aggregates and sends it to the corresponding RN. The RN determines the optimal inter-cluster routing based on the minimum spanning tree method to transmit data to the BS. Figure 4 illustrates the process of the protocol operating.

### Cluster head’s and relay node’s selection based on multi-strategy fusion snake optimizer

At present, most of the mataheuristic algorithms used for WSN cluster routing problems are easily prone to local optimum, which leads to uneven network energy consumption. Consequently, in order to overcome these issues, a new technique must be developed. In this paper, to select the best CH and RN for WSN, a clustering technique based on the multi-strategy fusion snake optimizer (MSSO) is presented. The descriptions of key parameters of MSSO are listed in Table 1.

#### Snake optimizer

Snake optimizer (SO) is a new meta-heuristic algorithm proposed by Fatma A.Hashim et al.^44^. inspired by the foraging and breeding behavior of snakes. Abundant optimization methods are hallmarks of SO, and it can maintain exploitation and exploration in control. Numerous research have employed SO to address various kinds of optimization issues^45,46^.

Like all metaheuristics algorithms, the initial population for the SO is generated by a random uniform distribution with the generation rule described in the following Eq. (20).

20 $$\begin{aligned} S_i=S_{m\dot{m}}+rand\times (S_{max}-S_{min}) \end{aligned}$$

where $S_i$ is a vector of length *Dim*,representing the position of the *i*-th individual in the population, *Dim* represents the problem dimension, *rand* denotes the random value between (0, 1), $S_{max}$ and $S_{min}$ denote the population’s maximum bounds and minimum bounds.

After obtaining the initial population, the population will be divided into male and female subpopulations. In general, the number of males and females is equal. Therefore, the number of female and male individuals can be calculated by Eq. (21).

21 $$\begin{aligned} N_m=N_f=\frac{N_{all}}{2} \end{aligned}$$

where $N_{all}$ is the total population size, $N_m$ and $N_f$ denote the number of male and female snakes, respectively.

In SO, The primary determinants of snake mating behavior are temperature and food quantity, and temperature *Temp* and food quantity *Q* can be defined by Eqs. (22) and (23).

22 $$\begin{aligned} Temp&=e^{-t/T}{} & {} \end{aligned}$$

23 $$\begin{aligned} Q&=c_1\times e^{(t-T)/T} \end{aligned}$$

where *t* denotes the current number of iterations, *T* denotes the total number of iterations, and $c_1$ is a fixed constant of 0.5.

SO uses preset thresholds $th_{Q}$ and $th_{T}$, where $th_{Q}$ = 0.25 and $th_{T}$ = 0.6, to detect if there is enough food and whether the temperature is high or low. The algorithm executes the exploration stage when $Q<th_{Q}$, meaning that the food is not enough. The snakes search for food by randomly selecting a location and updating it. Therefore, Eqs. (24) and (25) can simulate the exploration phase.

24 $$\begin{aligned} S_i^m(t+1)&=S_{rand}^m(t)\pm c_2\times e^{-f_{rand}^m/f_i^m}\times S_i \end{aligned}$$

25 $$\begin{aligned} S_{i}^{f}(t+1)&=S_{rand}^{f}(t)\pm c_{2}\times e^{-f_{rand}^{f}/f_{i}^{f}}\times S_{i} \end{aligned}$$

where $S_i^m$ denotes the position of the *i*-th male snake and $S_{rand}^m$ is the position of the random male snake. $f_{rand}^m$ is the fitness of the $S_{rand}^m$, and $f_i^m$ is the fitness of the$S_i^m$. $S_i^f$ denotes the position of the *i*-th female snake, and $S_{rand}^f$ denotes the position of the random female snake. $f_{rand}^f$ is the fitness of the $S_{rand}^f$, and $f_i^f$ is the fitness of the $S_i^f$. $c_2$ is a fixed constant of 0.05.

When there is enough food, the algorithm enters the exploitation stage. When $Q>th_{Q}$ and $Temp>th_{T}$, the temperature is high, the snake only eats food but can’t mate. The process can be represented by Eq. (26).

26 $$\begin{aligned} S_i^{m,f}\left( t+1\right) =S_{food}\pm c_3\times Temp\times rand\times \left( S_{food}-S_i^{m,f}\left( t\right) \right) \end{aligned}$$

where $S_i^{m,f}$ is the position of the *i*-th male or female individual, $S_{food}$ is the position of the optimal individual, and $c_3$ is a fixed constant of 2.

When $Q>th_{Q}$ and also $Temp\le th_T$, the temperature is low,the snake will into the mating part, and the same-sex individuals engage in intra-sexual competition for exclusive mating opportunities with the opposite sex. The winning individuals can give preference to mate, so there is a fighting mode and a mating mode in the mating part, and the fighting mode is represented by Eqs. (27) and (28), respectively.

27 $$\begin{aligned} S_i^m(t+1)&=S_i^m(t)+c_3\times e^{-f_{best}^f/f_i}\times rand\times \left( Q\times S_{best}^f-S_i^m(t)\right) \end{aligned}$$

28 $$\begin{aligned} S_i^f\left( t+1\right)&=S_i^f\left( t\right) +c_3\times e^{-f_{best}^m/f_i}\times rand\times \left( Q\times S_{best}^m-S_i^f\left( t\right) \right) \end{aligned}$$

where $S_i^m$ denotes the position of the *i*-th male snake, $S_{best}^f$ denotes the position of the optimal individual in the female snake, $S_i^f$denotes the position of the *i*-th female snake, $S_{best}^m$ is the position of the optimal individual in the male snake, $f_{best}^f$ is the fitness of the best individual of female group, $f_{best}^m$ is the fitness of the best individual of male group, and $f_i$ is the fitness of the *i*-th individual.

The mating pattern is represented by Eqs. (29) and (30), respectively.

29 $$\begin{aligned} S_i^m\left( t+1\right)&=S_i^m\left( t\right) +c_3\times e^{-m_i^f/f_i^m}\times rand\times \left( Q\times S_i^f\left( t\right) -S_i^m\left( t\right) \right) \end{aligned}$$

30 $$\begin{aligned} S_i^f\left( t+1\right)&=S_i^f\left( t\right) +c_3\times e^{-m_i^m/f_i^f}\times rand\times \left( Q\times S_i^m\left( t\right) -S_i^f\left( t\right) \right){} & {} \end{aligned}$$

where $S_i^m$ is the position of the *i*-th individual in the male snake, $S_i^f$is the position of the *i*-th individual in the female snake. $m_i^m$ and $m_i^f$ denote the fitness of the *i*-th individual male snake and female snake in the mating pattern.

When mating behavior is completed, the female snakes will lay and incubate eggs to obtain new snakes and select worst male and female and replace them. The replacement process is represented by Eqs. (31) and (32).

31 $$\begin{aligned} S_{worst}^m&=S_{min}+rand\times (S_{max}-S_{min}) \end{aligned}$$

32 $$\begin{aligned} S_{worst}^f&=S_{min}+rand\times (S_{max}-S_{min}) \end{aligned}$$

where $S_{worst}^m$is the worst individual among males and $S_{worst}^f$is the worst individual among females.

The pseudocode of the snake optimizer algorithm is shown in Algorithm 1.

**Algorithm 1:**
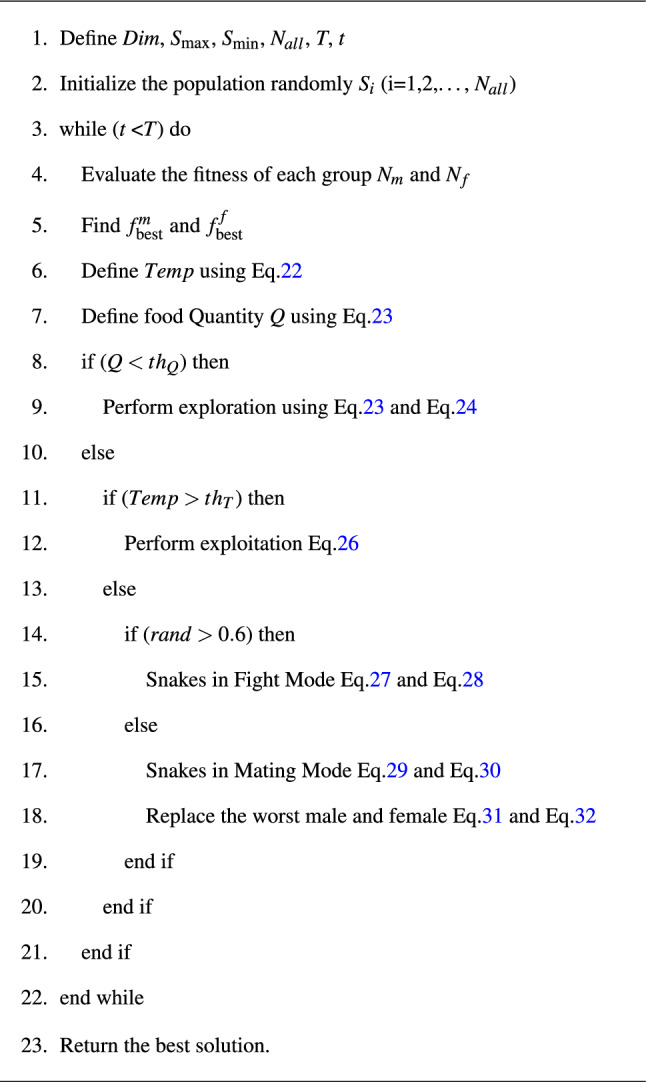
Snake optimizer (SO).

#### Multi-strategy fusion snake optimizer

Due to its unique algorithm structure, SO has shown promise in tackling optimization issues; nonetheless, it also suffers from slower convergence and poor population diversity. This section proposed the MSSO algorithm, which with three enhancement strategies based on the shortcomings of SO.

##### Dynamic parameter updating

There are several fixed-value parameters in the original SO, which has an impact on the algorithm’s optimization process. As a result, in this work, $c_1$, $c_2$, and $c_3$ in SO are dynamically updated^47^. The precise equation is as follows:

33 $$\begin{aligned} c_1^{new}&=c_1+\frac{1}{10}\times \cos \left( \textit{rand}^4\times \frac{\pi }{2}\right) \end{aligned}$$

34 $$\begin{aligned} c_2^{new}&=c_2+\frac{1}{1000}\times \cos \left( rand^4\times \frac{\pi }{2}\right) \ \end{aligned}$$

35 $$\begin{aligned} c_3^{new}&=c_3-2\times \sin \left( \left( \frac{t}{T}\right) ^4\times \frac{\pi }{2}\right) \end{aligned}$$

Upgrades to $c_1$, $c_2$, and $c_3$ can improve the algorithm’s ability to jump out of the local optimum, achieve a random transition between exploration and exploitation, and speed up the algorithm’s convergence, respectively, all contributing to its improved optimization performance.

##### Adaptive alpha mutation

**Figure 5:**
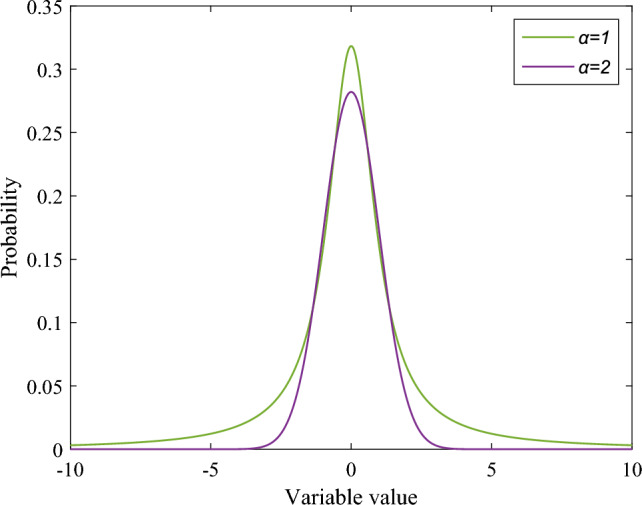
Plot of probability distribution function corresponding to different $\alpha$.

Alpha stable distribution is the limit distribution of infinitely many independent and identically distributed random variables that may contain infinite variance. It is a large class of widely representative random distribution models. Its probability density distribution function can be defined by the continuous Fourier transform of its characteristic function:

36 $$\begin{aligned} f\left( y;\alpha ,\theta ,\gamma \right) =\frac{1}{2\pi }\int _{-\infty }^{\infty }\varphi (x)e^{-ity}\textrm{d}x\ \end{aligned}$$

where $\varphi (x)$ is the characteristic function of the stable distribution, and the calculation method is as,

37 $$\begin{aligned} \varphi (x)=\exp \left[ i\delta x-\mid \gamma x\mid ^\alpha B_{x,\alpha }\right] \end{aligned}$$

38 $$\begin{aligned} B_{.x,\alpha }={\left\{ \begin{array}{ll}1-i\theta sgn(x)tan\biggl (\frac{\pi \alpha }{2}\biggr ),\alpha \ne 1\\ \\ 1+i\theta sgn(x)\frac{2}{\pi }\textrm{ln}(t),\quad \alpha =1\end{array}\right. } \end{aligned}$$

where $\gamma$ is the scale parameter,and the effect is similar to variance,The symmetric parameter $\theta$ determines the inclination of the distribution. $\delta$ is the position parameter,which corresponds to the mean and median of the stable distribution. In this paper, $\theta =0$, $\gamma =1$, $\delta =0$.The characteristic index, represented by $\alpha$, defines the random process’s level of impulse. The stronger the impulse and longer the tail, the smaller the $\alpha$ value, indicating a higher chance of random variables being away from the center. When $\alpha =1$, $\theta =0$, $\gamma =1$, $\delta =0$, the alpha stable distribution is Cauchy distribution ; when $\alpha =2$, $\theta =0$, $\gamma =1$, $\delta =0$, the alpha stable distribution is the Gaussian distribution. Figure 5 shows the characteristics of the probability distribution function corresponding to different $\alpha$ values.

Currently, the majority of research only looks at the single mutation modes of Cauchy and Gaussian mutation, and a few cases of mixed use of the two mutation modes do not fully meet the performance requirements of the algorithm in different periods. Consequently, the optimal individual’s position in the male and female populations of SO is considered to be disturbed by the random vector produced by the alpha stable distribution in this study, and the position before and after the disturbance is chosen using the greedy method.

39 $$\begin{aligned} S_{new\_best}^f&=S_{best}^f+S_{best}^f*S(\alpha ) \end{aligned}$$

40 $$\begin{aligned} S_{new\_best}^m&=S_{best}^m+S_{best}^m*S(\alpha ) \end{aligned}$$

41 $$\begin{aligned} \alpha&=2-\exp (-tan(\frac{t}{T})^\wedge 10) \end{aligned}$$

where $S(\alpha )$ is the random disturbance step size generated by the alpha stable distribution, $*$ represents the point multiplication operation, $\alpha$ is the adaptive characteristic index, which is used to control the variation ability of the algorithm in different periods, $S_{best}^m$ and $S_{best}^f$ are the optimal individuals in the female and male populations after the disturbance. When Eq. (41) and Fig. 5 are combined, it is evident that $\alpha$ is tiny in the algorithm’s early and intermediate phases. The mutation is currently approaching Cauchy mutation, which can enhance the algorithm’s capacity for global search and is easily capable of producing big extreme results and outliers. The range of mutation values rapidly narrows and approaches the Gaussian mutation method in the middle and late phases of the iteration, when $\alpha$ achieves nonlinear growth. This can enhance the algorithm’s search accuracy later on. Ultimately, the algorithm’s performance criteria are met in various times by means of the adaptive adjustment of $\alpha$. This method is referred to as the adaptive alpha mutation strategy in this research.

##### Bi-directional search optimization

Bi-directional search optimization (BDS) is a strategy that utilizes the best and worst individuals to explore within the search area. It is designed to keep the algorithm away from the worst individual while staying close to the best one. During the exploration stage, the original SO merely used random search, which had low search efficiency and significant uncertainty. In order to make the algorithm approach the optimal value while utilizing the entire search area and accelerating the speed of convergence, this study employs the BDS technique in conjunction with the optimal solution following adaptive alpha mutation disturbance. The equation is expressed as follows :

42 $$\begin{aligned} S_i^m\left( t+1\right)&=S_i^m\left( t\right) +r_1\times \left( S_{new\_best}^m-S_i^m\left( t\right) \right) -r_2\times \left( S_{worst}^m-S_i^m\left( t\right) \right) \end{aligned}$$

43 $$\begin{aligned} S_i^m\left( t+1\right)&=S_i^m\left( t\right) +r_1\times \left( S_{new\_best}^m-S_i^m\left( t\right) \right) -r_2\times \left( S_{worst}^m-S_i^m\left( t\right) \right) \end{aligned}$$

where $r_1$, $r_2$ are random numbers between 0 and 1.

The steps of using the MSSO to select CH or RN are as follows :

##### Step 1 input parameters

Input the algorithm parameters including $N_{all}$, $S_{max}$, $S_{min}$, *T*, $th_{Q}$, $th_{T}$, $c_1$, $c_2$, and $c_3$, *K*, $\omega$, $\lambda$.

##### Step 2 population initialization

The population individuals are initialized according to the Eq. (20). At this point, $S_{max}$ stands for the network’s boundary length, $S_{min}$ is 0, and the position data of the selected node set is represented by a snake individual in the population. Assuming that *K* nodes need to be selected as CH, let $S_i=\left[ S_{i,1}(t),S_{i,2}(t),...,S_{i,k}(t)\right]$ represent the *i*-th snake individual, where each component $S_{i,k}(t)=(x_{ik}(t),y_{ik}(t))$ represents the abscissa and ordinate of the sensor node selected as the CH. Then the snake individual selected for the CH can be expressed as : $S_i=\left[ (x_{i1}(t),y_{i2}(t)),(x_{i2}(t),y_{i2}(t)),...,(x_{iK}(t),y_{iK}(t))\right]$, the problem dimension Dim at this time is 2*K*, and the representation of the snake individual when selecting the RN is consistent with the CH selection.

Position mapping processing is required in order to make the position information in the snake individual match the actual coordinates of the nodes in the network. The position information in the snake individual is mapped to the sensor node closest to the network coordinate when choosing the CH. This node then becomes the CH to resolve the discrepancy between the individual position and the actual surviving node position caused by the discrete distribution of the network nodes. When executing location mapping, it is crucial to first ascertain whether the present closest node has been recognized as a CH in order to prevent the scenario where a node is mapped by numerous locations. If the present closest node has been mapped to a CH, the next closest node is selected for the location in turn. The location mapping method when selecting the RN is the same as when selecting the CH.

It is worth mentioning that when selecting CH, it can be seen from the Eq. (10) that the CH set with a more balanced location distribution in the network will have a better fitness value. Therefore, when using the MSSO to select cluster heads, the FCM algorithm is applied in the population initialization stage. The surviving nodes in the network are clustered once, and the obtained cluster center coordinates are embedded into the population to replace a random individual in the population, so as to achieve the purpose of accelerating the convergence of the algorithm.

After generating the initial population, the population is divided into two parts : male and female, and the algorithm iteration is started. Let *t* = 1;

##### Step 3 fitness evaluation

When selecting the CH, the fitness of the population is evaluated according to the objective function defined in Eq. (12). When selecting the RN, the fitness of the population is evaluated according to the objective function defined in Eq. (19). The population’s individual is better the smaller the objective function’s value. Find out the optimal individual in the female population and the male population.

##### Step 4 population location update

Firstly, the algorithm parameters are updated according to Eqs. (33) and (35), and then the food quantity and temperature are calculated according to Eqs. (22) and (23).

If $Q<th_{Q}$, the snake is in foraging mode, the search space is explored according to the BDS strategy using Eqs. (42) and (43) instead of the random walk strategy in the original SO.

If $Q>th_{Q}$ and ,the snake only eats food and does not go into mating, the position is updated according to Eq. (26).

If $Q>th_{Q}$ and also $Temp\le th_T$,when,the snake is in fight mode, and the position is updated according to Eqs. (27) and (28). Otherwise, the snake is in mating mode, and the position is updated according to Eqs. (29) and (30). And update the position of the worst individual in the male and female population.

When the population update is completed, the optimal individuals in the male and female population is adaptively disturbed by adaptive alpha mutation according to Eqs. (39) and (41). According to the greedy strategy, the better solutions before and after the disturbance are retained, and the position information in the population is mapped to the actual nodes in the network according to the position mapping strategy.

##### Step 5 determine whether the iteration is terminated

If *t* < *T*, then return to step 3, otherwise, the algorithm ends, and the best CH set or the best RN set are output.

### Inter cluster routing between relay nodes

After selecting the optimal RN set, this paper will determine whether the RN transmits data to the BS in a single-hop or multi-hop manner according to the dis tance *d* between the RN and the BS. If $d\ge d_0$ , it is evident from Eq. (2) that the data transmission node’s energy consumption is excessive, and the data should be transmitted in a multi-hop manner, in the absence of such, single-hop transmission is adopted^48^. For the purpose to accomplish the goals of reducing network energy consumption and balancing the consumption of forwarding nodes, this paper employs the method of constructing minimum spanning tree to generate the shortest path of multi-hop transmission of relay nodes, taking into account the residual energy and directionality of nodes.

The routing between RN is built a weighted connected graph *G* = (*V*, *E*), where *V* is the set of all RN and *E* is the set of edges connecting any two nodes in *V*. It starts with the BS as the root of the tree, abstracts each forwarding node as a point, and connects them with edges. The best route from a RN to the BS must be found by considering the remaining energy and distance between neighboring RN.

Assuming that the $RN_{i}$ is the starting point, it is required to search the next hop node passes to the BS. Suppose that $RN_{j}$ is an adjacent relay node of $RN_{i}$,it is determined whether or not $RN_{j}$ can be utilized as $RN_{i}$’s next hop node by comparing the edge weights of the two nodes. $w_{i,j}$ is the weight, which is based on the remaining energy and distance between the two nodes. If the distance between the $RN_{j}$ and the BS is more than or equal to the distance between the $RN_{i}$ and the BS, then $RN_{j}$ shouldn’t be the next hop node, in which case $w_{i,j}$ = ${\infty }$. Otherwise, $w_{i,j}$ is calculated by the distance between two nodes and the residual energy. The calculation method is as follows :

44 $$\begin{aligned} w_{i,j}=\frac{E_{TX}(l,d_{i,j})}{E_{RN}^{r}(i)E_{RN}^{r}(j)} \end{aligned}$$

where $d_{i,j}$ is the distance between $RN_{i}$ and $RN_{j}$, $E_{RN}^r(i)$ and $E_{RN}^r(j)$ represent the residual energy of $RN_{i}$ and $RN_{j}$. It is evident that a smaller distance between the two nodes and a higher residual energy will result in a smaller weight between the two nodes and a higher likelihood that $RN_{j}$ will be chosen as $RN_{i}$’s next hop node. The relay node with the lowest related weight is chosen as the next hop node if there are several adjacent relay nodes. The following is the precise procedure for the minimum spanning tree-based inter-cluster routing establishment approach. The base station, BS, is added to *V* as the tree’s root node in the weighted connected graph *G* = (*V*, *E*) that was previously formed. $W_{1}$ records the weight set of the edges generated by the relay node to be selected and its neighbor nodes, $W_{2}$ records the weight set of the edges in the minimum spanning tree, and *U* records the node set of the minimum spanning tree.

- *Step 1* $W_{1}$ and $W_{2}$ are initially empty, and the root node BS is added to *U*.
- *Step 2* The distance $d_{i,BS}$ from the node $RN_{i}$ to the BS in *V* is calculated in turn, and compared with the preset threshold distance .If $d_{i,BS}<d_{0},$, then $RN_{i}$ use a single hop to transmit data to the base station, add $RN_{i}$ to *U*, set the edge weight $w_{i,BS}$ = 0 between I and BS, and add $w_{i,BS}$ to $W_{1}$, go to step 5. Otherwise, go to step 3 and establish a multi-hop path from $RN_{i}$ to BS.
- *Step 3* The weights of $RN_{i}$ to all other relay nodes are calculated and added to$W_{1}$.
- *Step 4* In $W_{1}$, the minimum weight $w_{i,k}$ is chosen. At this time, the distance from $RN_{i}$ to BS is $d_{i,BS}$, the distance from $RN_{k}$ to BS is $d_{k,BS}$, and the distance from $RN_{i}$ to $RN_{k}$ is $d_{i,k}$. If $d_{i,BS}<d_{i,k}$, then $RN_{k}$ is not in the right direction for $RN_{i}$, or $E_{TX}(l,d_{i,BS})<E_{TX}(l,d_{i,k})+E_{TX}(l,d_{k,BS})$, then the overhead of forwarding data from $RN_{i}$ to BS through $RN_{k}$ is greater. In this case, $RN_{i}$ will send data to BS directly, then add node $RN_{i}$ to *U*, set the edge weight $w_{i,BS}$ = 0 between $RN_{i}$ and BS, add $w_{i,BS}$ to $W_{2}$, and set $W_{1}$ to empty. Otherwise, the node $RN_{k}$ is added to *U*, and $w_{i,k}$ is added to $W_{2}$ and set $W_{1}$ to empty.
- *Step 5* If *U* = *V*, end the search and go to step 6 ; otherwise, go to Step 2.
- *Step 6* When there is a weight with a value of 0 in $W_{2}$ and the latter node in the weight’s corresponding edge is BS, the previous node in the corresponding edge is output as a single-hop node; otherwise, the latter node serves as the next hop node and the previous node serves as the starting point. To build a multi-hop path output, keep looking for the next hop node until it is BS.

### Time complexity analysis

This paper’s protocol is run in rounds, with *Maxround* serving as the maximum running round.The time complexity analysis of the protocol discussed in this study can also be divided into two parts, since each round of the protocol is separated into steps for cluster setup and data transmission.

In the cluster set-up stage, assuming that *K* clusters need to be formed, it is necessary to use MSSO to select reasonable *K* CH and RN respectively. The time complexity of the original SO is $O(T\times N_{all}\times Dim)$. For MSSO, the complexity of the algorithm increased by dynamically updating the parameters is *O*(*T*). The BDS strategy does not increase the complexity on the basis of the original algorithm because it replaces the random walk mechanism in the original SO. The complexity of the algorithm increased by adaptive alpha mutation is $O(T\times Dim)$, and the time complexity of MSSO is $O(T\times N_{all}\times Dim)+O(T)+O(T\times Dim)$. The time complexity of the final MSSO is $O(T\times N_{all}\times \bar{Dim})$ since it only takes the primary item into account.

When using MSSO to select *K* CH or RN, the above analysis shows that the problem dimension *Dim* = 2*K*, so the time complexity of using MSSO to select CH or RN can be expressed as $O(T\times N_{all}\times 2K)$, then in the cluster set-up stage, the time complexity of the proposed protocol is $O(\textit{Maxround}\times T\times N_{all}\times 2K)+O(\textit{Maxround}\times T\times N_{all}\times 2K)$, similarly, the complexity can be simplified to $O(Maxround\times T\times N_{all}\times K)$.

In the data transmission phase, the time complexity required to determine the optimal transmission route from *K* relay nodes to BS by using the minimum spanning tree is $O(K\times K)$, so the time complexity of the protocol operation in the data transmission phase is $O(Maxround\times K\times K)$.

In conclusion, the time complexity of the protocol proposed in this study is $O(Maxround\times T\times N_{all}\times K)+O(Maxround\times K\times K)$, and because the value of *K* is smaller than that of $T\times N_{all}$. Therefore, the final time complexity is $O(Maxround\times T\times N_{all}\times K)$.

## Experimental results and analysis

In this section, the proposed protocol is compared with several protocols such as LEACH^12^, ESO^35^, EEWC^22^, GWO^29^ and EECHS-ISSADE^31^. A comparison of the network lifetime, node death, residual energy, energy balance, throughput, and other parameters shows the viability and efficiency of the proposed protocol. The experiments are conducted using MATLAB 2022a software on a computer equipped with an Intel (R) Core (TM) i5-8300H CPU, 8 GB RAM, and the Windows 10 operating system.

**Table 2:** Model setting.

Parameter	Value
Initial energy	0.5 J
$\delta _{elec}$	50 nJ/bit
$\varepsilon _{fs}$	10 pJ/bit/m$^{2}$
$\varepsilon _{mp}$	0.0013 pJ/bit/m$^{4}$
$\varepsilon _{da}$	5 nJ/bit
$d_0$	87.7 m
Packet length	4000 bits
Control packet length	200 bits
*p*	0.05
MAC protocol	IEEE 802.15.4

The sensor nodes in this study have a normal random distribution, and the network topology is clustered and homogeneous. The BS is arranged outside of the network to better reflect the real scenario. In the simulation experiments, a carefully designed traffic pattern was employed to replicate the typical monitoring application in environmental sensing. The sensor nodes were configured to generate data packets periodically at fixed time intervals. This mode simulates the conventional sensing and reporting behavior of sensor nodes in response to environmental changes, and the communication parameter settings are shown in Table 2. Table 3 displays the MSSO algorithm’s parameter settings, while the comparison algorithm’s parameter values align with the original literature.

**Table 3:** Parameter of MSSO.

Parameter	Value
*T*	30
$N_{all}$	30
$th_{Q}$	0.25
$th_{T}$	0.6
$c_1$	0.5
$c_2$	0.05
$c_3$	2
$\theta$,$\gamma$,and $\delta$	0.1 and 0

Simultaneously, two network scenarios are utilized as examples to illustrate the scalability and stability of the proposed protocol. The experimental parameter settings are shown in Table 4. This work employs the Monte Carlo approach to provide more thorough and precise analysis while also improving the experimental data’ statistical reliability and unpredictability. By averaging the data from 100 consecutive experiments, the findings and conclusions of the experiment are obtained. This paper sets the weight $\omega$ and $\lambda$ value of the objective function in the Eq. (12) and (19) to 0.5 after conducting several comparative experiments to better assess the effectiveness of the proposed protocol.

**Table 4:** Experimental scenario settings.

	Scenario 1	Scenario 2
Number of nodes	100	200
Field dimension	[100 m,100 m]	[200 m, 200 m]
BS location	(50 m, 250 m)	(100 m, 500 m)

### Analysis of network lifetime

**Figure 6:**
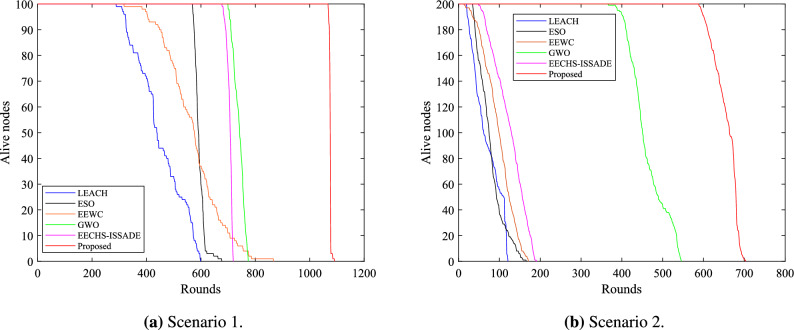
Number of alive nodes in network in different scenarios.

**Figure 7:**
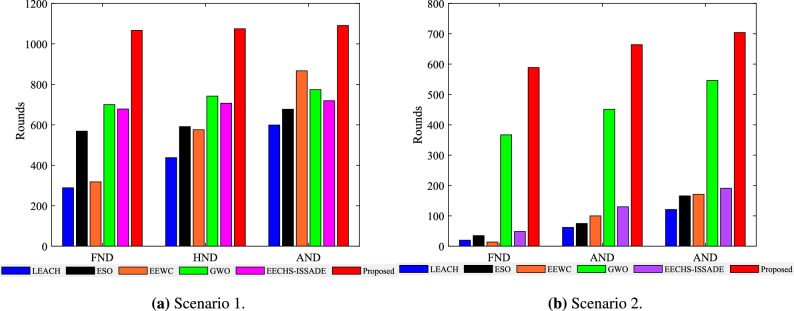
The time when a specified number of dead nodes appear in network in different scenarios.

The network lifetime is defined as the rounds of death of all nodes in the network, and the network stability period is defined as the running rounds of the first death node. Figure 6 depicts the survival of nodes in the network when each protocol runs in different scenarios. A columnar distribution map representing the first node death (FND), half of node death (HND), and all node death (AND) rounds for each protocol in different scenarios is shown in Fig. 7.

In scenario 1,the proposed method runs 1091 rounds, while the LEACH, ESO, EEWC, GWO, and EECHS-ISSADE protocols run 599, 677, 867, 774, 719 rounds, respectively, when all nodes are dead. In comparison to other protocols, the network lifetime rises by 82.14%, 61.15%, 25.84%, 40.96%, and 51.73%, respectively. When the first dead node occurs, the LEACH, ESO, EEWC, GWO, and EECHS-ISSADE protocols run 289, 569, 318, 100, 678 rounds respectively, while this protocol runs 1067 rounds. The network stability cycle increases by 269.20 %, 87.52 %, 235.54 %, 52.43 % and 57.38 % respectively compared with other protocols.

**Figure 8:**
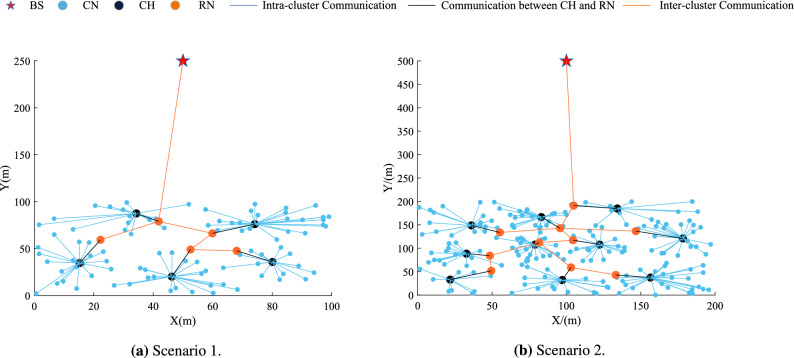
Example diagram of the operation of the proposed protocol in different scenarios.

The proposed protocol runs 704 rounds in scenario 2, while the LEACH, ESO, EEWC, GWO, and EECHS-ISSADE protocols run 121, 166, 171, 546, 191 rounds, respectively, when all nodes are dead. The network lifetime increases by 481.82%, 324.10%, 311.70%, 28.94%, and 268.57%, correspondingly, in comparison to other protocols. When the first dead node appears, LEACH, ESO, EEWC, GWO and EECHS-ISSADE protocols have run 20, 35, 14, 367 and 49 rounds respectively, while the protocol in this paper has run 589 rounds. The network stability period has increased by 2845.00 %, 1582.86 %, 4107.14 %, 60.49 %, and 1102.04%, in comparison to other protocols.

LEACH, ESO, EEWC, and EECHS-ISSADE protocols expire earlier in scenario 2 because nodes require more energy for transmitting data as network scale and BS distance increase. In this case,the algorithm used to select CH must comply with stricter criteria for efficiency and rationality. In addition, to reduce cluster head overhead, inter-cluster routing must also be planned. Although GWO designs additional inter-cluster routing to save energy and prolong the network lifetime and stable period, with the increase of network scale, more cluster heads must be chosen, which means that the dimension of the optimization problem will also increase, the algorithm may fall into local optimum at this time, and the CH load is not considered in the protocol, resulting in limited performance improvement. In contrast, the protocol put forward in this paper can significantly guarantee the network operates stably in both scenarios, particularly in the event that all nodes in other protocols dies and the network keeps operating as usual. Figure 8 shows the network topology of a certain round in the stable period of the proposed protocol. Benefiting from the reasonable selection of CH and RN based on MSSO and the determination of efficient inter-cluster routing, the proposed protocol has both high efficiency and good scalability in prolonging the network lifetime.

### Analysis of residual energy

**Figure 9:**
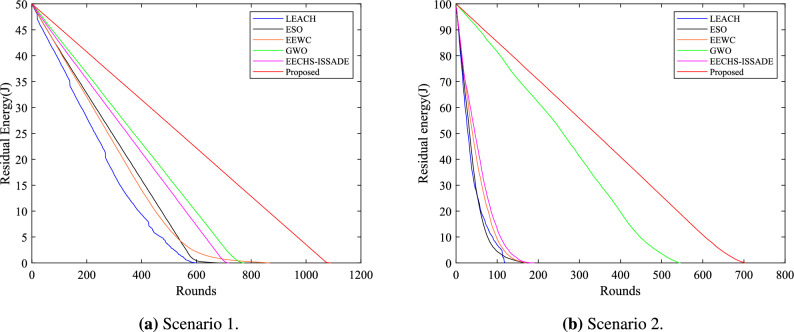
Residual energy in network in different scenarios.

The residual energy of the network for each protocol in different scenarios is shown in Fig. 9. LEACH protocol consumes the energy of all nodes first due to the randomness of CH selection. ESO, EEWC and EECHS-ISSAD introduce different metaheuristic algorithms to optimize CH selection respectively. In scenario 1, they can have more energy in the same running rounds compared to the LEACH protocol, while in scenario 2, the effect is less evident. GWO takes inter-cluster routing into account while introducing metaheuristic algorithm to select the best CH, and performs well in different scenarios. However, the network’s residual energy level is still constrained because of the algorithm’s sensitivity to the initial clustering’s construction and its incapacity to optimize efficiently due to limit of iterations of metaheuristic algorithm. The protocol proposed in this research could equalize the algorithm’s exploration and exploitation capabilities in a finite number of iterations by incorporating BDS strategy, adaptive alpha mutation and dynamic updating mechanism. In different scenarios, it can rapidly recognize excellent CH and RN while maintaining the convergence speed and avoiding local optimum as much as possible. Furthermore, the population’s sensitivity to the initial clustering center is mitigated by the introduction of FCM algorithm, and each RN’s optimal transmission path is established through the minimum spanning tree. Therefore, when operating, the proposed protocol can consistently maintain a higher residual energy level than previous protocols.

**Figure 10:**
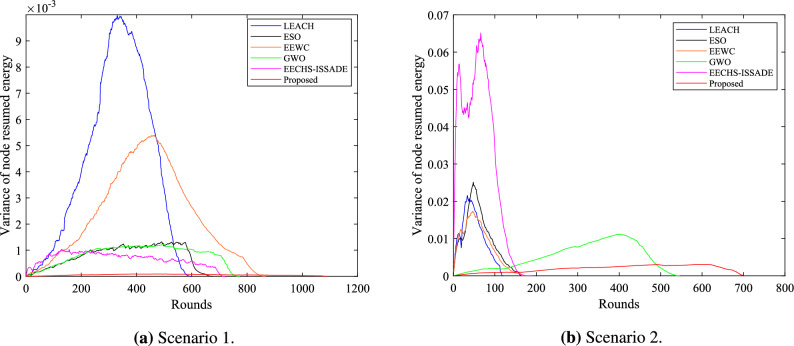
Variance of node residual energy in network in different scenarios.

**Figure 11:**
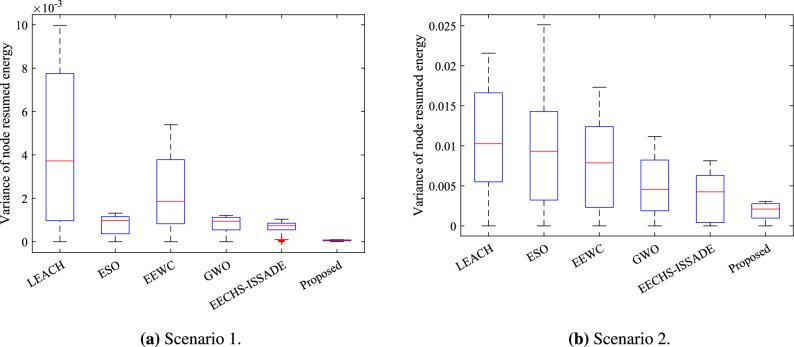
Box plot of variance of node residual energy in network in different scenarios.

In order to further verify the balance of the residual energy of the nodes during the operation of each protocol, this study makes statistics on the variance of the residual energy of the nodes in the network during the operation of each protocol in different scenarios. The results are shown in Figs. 10 and 11.

In scenario 1, the variance of the residual energy of the nodes corresponding to the LEACH protocol is the largest, indicating that the energy consumption of the nodes is not balanced during the operation of the protocol, and some nodes have excessive energy consumption, which directly leads to the premature death of some nodes. Even though EEWC clearly lengthens the network lifetime, the figure shows that residual energy levels among nodes still differ significantly, which is also the reason why EEWC fails to effectively extend the network stability period. ESO, EECHS-ISSADE and GWO control the residual energy level difference of nodes in a smaller range in scenario 1, which can better allocate the energy consumption in the network to different nodes on average compared with LEACH, but scenario 2 exhibits significantly varied node residual energies of these protocols, and an even higher variance in the ESO than the LEACH protocol during operation. This might be some nodes become CH more frequently than others because ESO fails to specify an acceptable objective function to select CH, which results in excessive energy consumption. Although GWO saves network energy to some extent in scenario 2, it may make some CH undertake more transmission tasks due to the selection of forwarding nodes for multi-hop transmission between CH, resulting in a large difference in residual energy of nodes.

In contrast, the proposed protocol can select excellent RN to share the data transmission task of CH in different scenarios, thereby balancing the CH’ energy consumption. At the same time, RN energy consumption is decreased because multi-hop routing between relay nodes reduces the possibility of RN transmitting data directly to the BS. Figures 10 and 11 show that, while maintaining the network’s overall energy as much as feasible, the residual energy of each node can always be kept at the same level during protocol operation.

### Analysis of energy consumption

**Figure 12:**
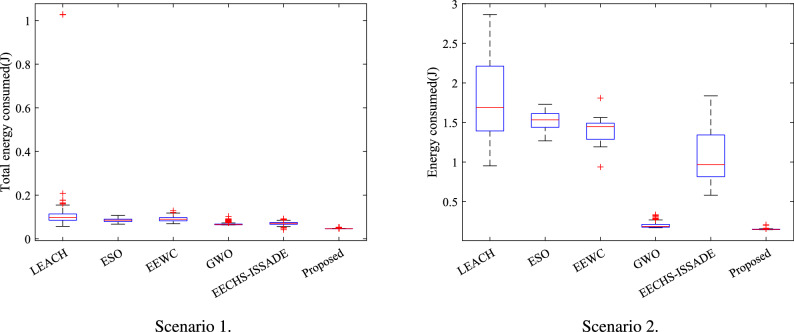
Box plot of total energy consumed in network in different scenarios.

The network structure will be damaged following the appearance of the first dead node, which will have an impact on the comparative analysis of the network’s overall energy consumption while the protocol is in operation. Consequently, the entire energy consumption of the network in every round of all protocols in different scenarios over their corresponding stable periods is statistically compared in this section. Tables 5 and 6 and Fig. 12 are used to display the results.

**Table 5:** The energy consumption of different protocols in the stable period in Scenario 1.

	Max	Min	Avg	Std
LEACH	1.0276	0.0567	0.1100	0.0969
ESO	0.1068	0.0668	0.0844	0.0079
EEWC	0.1287	0.0686	0.0905	0.0109
GWO	0.1206	0.0630	0.0670	0.0055
EECHS-ISSADE	0.0914	0.0411	0.0708	0.0057
Proposed	0.0526	0.0457	0.0465	0.0011

**Table 6:** The energy consumption of different protocols in the stable period in Scenario 2.

	Max	Min	Avg	Std
LEACH	2.8642	0.9519	1.7846	0.5195
ESO	1.7296	1.2677	1.5143	0.1177
EEWC	1.8084	0.9375	1.4015	0.2006
GWO	0.3323	0.1697	0.1992	0.0328
EECHS-ISSADE	1.8362	0.5798	1.0698	0.3200
Proposed	0.2013	0.1435	0.1461	0.0082

As combined with Fig. 12 and Tables 5 and 6, it is evident that in Scenario 1, the LEACH protocol may, in certain instances, consume a substantial amount of the network’s total energy during the stable period. The maximum total energy consumption of each round of the network is 1.0276 J, and the minimum energy consumption of each round of the network is 0.0567 J, at this time, the energy consumed by the protocol operation is less. The final average total energy consumption of each round of the network is 0.1100 J. In the steady phase, ESO, EEWC, GWO, and EECHS-ISSADE use less energy per round than LEACH does. The average total energy consumption of each round of the network is 0.0844 J, 0.0905 J, 0.0670 J and 0.0708 J. Simultaneously, it avoids the occurrence of excessive consumption of energy. The maximum energy consumption of each round of the network is 0.1068 J, 0.1287 J, 0.1206 J and 0.0914 J, respectively. The proposed protocol can reduce the average energy consumption of each round of the network to 0.0465 J in a stable period, which is 58.64 %, 44.97 %, 63.87 %, 30.59 % and 34.33 % lower than LEACH, ESO, EEWC, GWO and EECHS-ISSADE,respectively. At the same time, the maximum energy consumption of each round of the network is only 0.0526 J. Compared with LEACH, ESO, EEWC, GWO and EECHS-ISSADE, it is reduced by 94.88 %, 56.48 %, 63.87 %, 61.48 % and 49.09 % respectively.

In scenario 2, the LEACH protocol still consumes a lot of energy in stable periods. The network’s maximum total energy consumption per round is 2.8642J, while the average total energy consumption per round is 1.7846J. The optimization of energy consumption by ESO,EEWC and EECHS-ISSADE has a restricted impact. The maximum energy consumption of each round of network is reduced to 1.7296 J, 1.8084 J and 1.8362 J, respectively. The average energy consumption of each round of network is reduced to 1.5143 J, 1.4015 J and 1.0698 J, respectively. GWO demonstrates some scalability in Scenario 2, which can reduce the maximum and average energy consumption to 0.3323 J and 0.1992 J, respectively. In this instance, the proposed protocol can reduce the maximum value of the total energy consumption of each round of the network to 0.2013 J, which is 92.97 %, 88.36 %, 88.86 %, 39.33 % and 89.04 % lower than LEACH, ESO, EEWC, GWO and EECHS-ISSADE. The average value of the total energy consumption of each round of the network is reduced to 0.1461 J, compared with LEACH, ESO, EEWC, GWO and EECHS-ISSADE, it is reduced by 91.87 %, 90.35 %, 89.58 %, 39.33 %, 26.64 % and 86.32 % respectively. The results of comprehensive Tables 5, 6 and Fig. 12 demonstrate that the proposed protocol has high efficiency and strong scalability in minimizing network energy consumption,with stable energy consumption in each round of network in different scenarios.

### Analysis of network throughput

**Figure 13:**
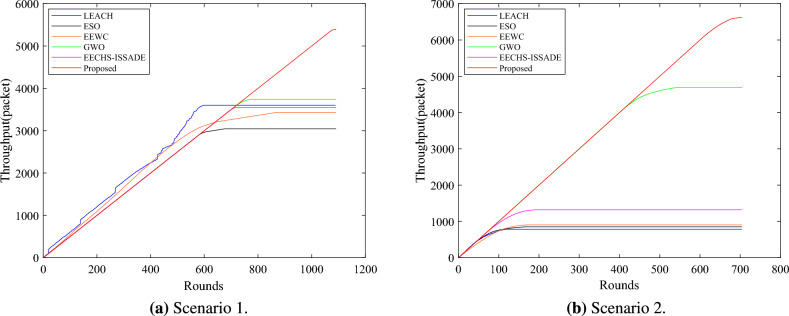
Throughput in network in different scenarios.

**Figure 14:**
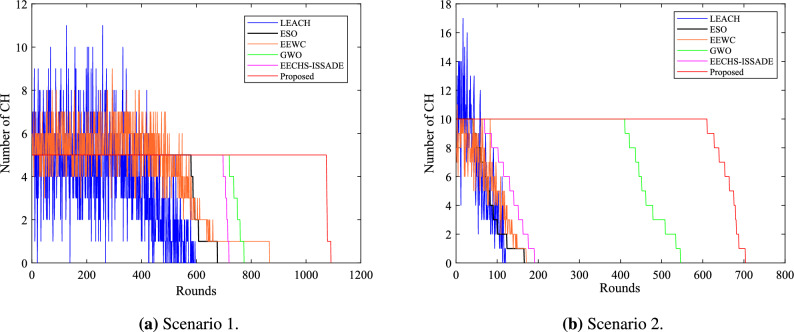
Number of CH in network in different scenarios.

The throughput is the amount of packets received by the BS before all the nodes die in the network. Figure 13 shows the network throughput comparison for each protocol operating in different scenarios. Since the network topology in this paper is a cluster structure, the CH collects and aggregates the data in the cluster and transmits the aggregated data packet to the BS.Therefore, the throughput is directly related to the number of CH in the network. Figure 14 shows the comparison of the number of CH when each protocol runs in different scenarios.

In scenario 1, the number of packets received by the BS in the LEACH protocol is 3599 when all the nodes in the network die, and the throughput maintains an advantage at the beginning. This is due to the fact that the number of CH varies greatly since the LEACH selects the CH at random depending on the CH probability. Additionally, the CH will not be selected in certain rounds. At this time,all sensor nodes will send their own sensing data directly to the BS in order to guarantee stable real-time monitoring of the region,resulting in a sharp increase in the number of packets received by the BS in this round, which is shown as a sharp increase in throughput in the Fig. 14. However, in this case, a large amount of energy storage is required, resulting in premature death of the node, and because the data perceived by the adjacent sensor nodes in the network is correlated, although the base station receives a large number of data packets, a considerable part of the data is redundant data, which is not helpful for network monitoring. In contrast to LEACH, EEWC limits the range of variation in the quantity of CH and prevents adverse occurrences that arise when there are no CH. The base station received 3429 data packets as a result of the extended network lifetime. However, when a large or small number of cluster heads are selected, the network may create a weak cluster, which could lead to some nodes dying early, restricting network throughput and making it difficult for the network to fully monitor the environment. Based on the probability of cluster heads, ESO, GWO, and EECHS-ISSADE have the ability to select a consistent number of cluster heads. Nevertheless, the final throughput is 3045, 3727, and 3546, respectively, because of the network lifetime limits. The proposed protocol can also select a stable number of CH in each round according to the CH probability. In addition, the BS received 5393 packets due to the benefits of network lifetime, and the throughput is 49.76 %, 77.14 %, 57.47 %, 44.77 %, and 51.98 % higher than LEACH, ESO, EEWC, GWO, and EECHS-ISSADE, respectively.

In scenario 2, due to the premature end of network lifetime, the throughput of LEACH, ESO, EEWC and EECHS-ISSADE is 784,852,904 and 1321 respectively, and the monitoring quality is poor. GWO has good scalability, and the throughput eventually reaches 4689, and the throughput eventually reaches 4689, while the proposed protocol can reach 6619, which is 743.11 %, 677.35 %, 632.87 %, 40.99 % and 400.15 % higher than LEACH, ESO, EEWC, GWO and EECHS-ISSADE respectively. It is obvious from the aforementioned results that the proposed protocol can improve network throughput in different scenarios. In addition, it can guarantee as much comprehensive real-time monitoring of the deployment region as feasible throughout the protocol’s operation and enhance the quality of the monitoring due to the extended network stability period.

## Conclusion

In this paper, an efficient cluster routing model that can accurately capture the requirements of the actual scene is proposed in order to save energy and extend the lifetime of the WSN. In addition, a new cluster routing protocol based on MSSO and minimum spanning tree is proposed to improve the energy efficiency of WSN and significantly prolong the longevity of the system. Moreover, the design of a new objective function for the MSSO greatly improves the rationality of cluster head and relay node selection. Furthermore, the embedding of FCM algorithm makes MMSO perform better in selecting cluster heads. Through comparative experiments, it is found that the proposed protocol can utilize network storage more effectively in the same network environment. Compared with several cluster routing protocols, it significantly reduces energy consumption and greatly improves network lifetime.

In the future research, we will discuss the performance of the proposed protocol in a wide range of scenarios, including underwater WSN, mine WSN and other complex environments. In addition, we will also focus on applying this method to special types of WSN, such as 3D WSN, mobile WSN, heterogeneous WSN or WSN with variable topology. At the same time, we will further investigate the stability of the proposed algorithm in more detail, which will help to ensure that it can provide more reliable service quality in practical applications, thus promoting the advancement and development of intelligent sensing technology in practical applications.

## Data Availability

All data generated or analyzed during this study are included in this published article.
